# Sponges from Plasma Treated Cellulose Nanofibers Grafted with Poly(ethylene glycol)methyl Ether Methacrylate

**DOI:** 10.3390/polym14214720

**Published:** 2022-11-04

**Authors:** Ioana Chiulan, Denis Mihaela Panaitescu, Andrada Serafim, Elena Ruxandra Radu, Gabriela Ioniţă, Valentin Rădiţoiu, Augusta Raluca Gabor, Cristian-Andi Nicolae, Marius Ghiurea, Dora Domnica Baciu

**Affiliations:** 1 Advanced Polymer Materials Group, University Politehnica of Bucharest, 011061 Bucharest, Romania; 2 National Institute for Research and Development in Chemistry and Petrochemistry, 202 Splaiul Independentei, 060021 Bucharest, Romania; 3 Institute of Physical Chemistry, Romanian Academy of Sciences, 202 Splaiul Independentei, 060021 Bucharest, Romania; 4 Cantacuzino National Medico-Military Institute for Research and Development, Bucharest, 103 Splaiul Independentei, 050096 Bucharest, Romania

**Keywords:** nanocellulose sponges, grafting, hydrophobicity, compression strength

## Abstract

In this work, cellulose nanofibers (CNF) were surface treated by plasma and grafted with poly(ethylene glycol)methyl ether methacrylate (PEGMMA) for increasing mechanical strength and hydrophobicity. The surface characteristics of the sponges were studied by scanning electron microscopy, micro-computed tomography, and Fourier transform infrared spectroscopy, which demonstrated successful surface modification. Plasma treatment applied to CNF suspension led to advanced defibrillation, and the resulting sponges (CNFpl) exhibited smaller wall thickness than CNF. The grafting of PEGMMA led to an increase in the wall thickness of the sponges and the number of larger pores when compared with the non-grafted counterparts. Sponges with increased hydrophobicity demonstrated by an almost 4 times increase in the water contact angle and better mechanical strength proved by 2.5 times increase in specific compression strength were obtained after PEGMMA grafting of plasma treated CNF. Cells cultivated on both neat and PEGMMA-grafted CNF sponges showed high viability (>99%). Remarkably, CNF grafted with PEGMMA showed better cell viability as compared with the untreated CNF sample; this difference is statistically significant (*p* < 0.05). In addition, the obtained sponges do not trigger an inflammatory response in macrophages, with TNF-α secretion by cells in contact with CNFpl, CNF-PEGMMA, and CNFpl-PEGMMA samples being lower than that observed for the CNF sample. All these results support the great potential of cellulose nanofibers surface treated by plasma and grafted with PEGMMA for biomedical applications.

## 1. Introduction

Nanocellulose is widely considered an advantageous material in a plethora of applications, as it is non-toxic, biodegradable, highly biocompatible, and offers excellent mechanical strength [1]. The high number of hydroxyl groups leads to strong hydrogen interactions between nanofibrils and plays a key role in the properties of nanocellulose. They are responsible for the gel-like structure of the suspensions with very low concentrations, demonstrating a high hydrophilic character, taking part in surface modification reactions and the synthesis of composites. For some specific applications, the high hydrophilicity of nanocellulose may be inconvenient; the absorbed water and moisture alter the mechanical strength and physical integrity of the cellulose-based materials [2] impart undesired wettability and speed up biodegradability. Additionally, the high hydrophilicity of nanocelluloses is also responsible for their tendency to agglomerate in synthetic polymers, most of them being characterized by high hydrophobicity.

The functionalization of native cellulose has been studied in order to achieve specific characteristics, such as improved solubility and mechanical strength or reduced hydrophilicity. Well-established procedures for surface chemical functionalization of cellulose include silylation [3], acetylation [4], oxidation [5], amination [6], sulfonation [7,8], and polymer grafting reactions. The cellulose modification with different monomers and macromonomers is a feasible way to change its physical and chemical characteristics and has been the subject of many works. It is often desirable that the grafted polymers impart cellulose with different surface characteristics without modifying its intrinsic properties, such as nanodimension, crystallinity, and mechanical properties [9]. In particular, the grafting of poly(ethylene oxide) moieties onto cellulose has been reported for different applications in thermal energy storage [10], for the development of gel polymer electrolyte [11], to prepare biosensors [12] and more flexible composites for packaging [13] or drug delivery systems [14]. In our earlier report, we identified and characterized 2,6,6-tetra-methylpiperidine-1-oxyl (TEMPO) oxidized cellulose nanofiber (TOCNF) as an efficient substrate to graft ethylene glycol methyl ether acrylate [15]. This chemical treatment has been proven to facilitate the grafting of acrylate oligomers on the surface of TOCNF compared with the original CNF, resulting in improved properties.

Several works have shown the advantages of treating cellulose with plasma [16,17,18]. Plasma is an environmentally-friendly method to modify the surface properties of cellulose [17]. It is ionized gas containing electrons, radicals, ions, neutral gas atoms, or molecules [19]. When cellulose films were activated with cold oxygen plasma at room temperature, this treatment induced ring opening and grafting of L-lactide, leading to increased interfacial compatibility in cellulose—poly(lactic acid) bi-layer laminates [17]. Similarly, the surface properties of cellulose films treated by dielectric barrier discharge (DBD) plasma at atmospheric pressure were changed in terms of roughness, hydrophilicity, and chemical composition, leading to enhanced cell adhesion and proliferation [18]. A new method to directly functionalize nanocellulose suspended in water is by being submerged in liquid plasma (SLP) [16]. SLP was able to modify the surface chemistry of nanocellulose, increasing its compatibility with poly(3-hydroxybutyrate) [16]. Therefore, SLP can be considered an effective, environmentally-friendly method to activate the surface of nanocellulose and an alternative to TEMPO oxidation.

One of the most popular monomers in the synthesis of smart materials is poly(ethylene glycol) methyl ether methacrylate (PEGMMA), which can respond to an environmental temperature by a reversible phase transition [20]. Considering the above, in the present study, cellulose nanofibers (CNF) were surface activated by a plasma jet immersed in the cellulose suspension and then grafted with PEGMMA. Thus, nanocellulose-PEGMMA sponges with tunable hydrophobicity and strength were obtained by applying a free radical graft polymerization technique. This unique method has the advantage of cumulating the eco-friendliness of plasma treatment with its double action of cellulose defibrillation and functionalization, having a notable effect on CNF characteristics and preventing clogging in subsequent grafting reactions.

## 2. Materials and Methods

### 2.1. Materials

Microcrystalline cellulose (MCC) with 20 μm mean particle size and 0.6 g/mL bulk density was purchased from Sigma Aldrich (Saint Louis, MO, USA). An aqueous suspension of 1 wt% MCC was passed through a microfluidizer processor (Microfluidics, Westwood, MA, USA), operating under high pressure. Ten cycles were performed in order to ensure the production of a homogenous suspension. Potassium persulfate (KPS) was supplied by Scharlau (Barcelona, Spain) and used as received. Poly(ethylene glycol)methyl ether methacrylate (Mn = 300) was supplied by Sigma Aldrich (Saint Louis, MO, USA) and was purified by passing through a short basic Al_2_O_3_ column prior to use.

### 2.2. Methods

#### 2.2.1. Plasma Treatment of Cellulose Suspension

Plasma treatment of cellulose suspension was prepared by dispersing CNF in bi-distilled water by ultrasonication for 1 h. The plasma treatment was carried out using a plasma torch source designed to work at atmospheric pressure in the open air [16]. The plasma jet was ignited in the open air (500 sccm argon, 150 W) and immersed in cellulose suspension for 30 min. The sample, denoted as CNFpl, was further used in the grafting reaction.

#### 2.2.2. Grafting Reaction

**Figure 1:**
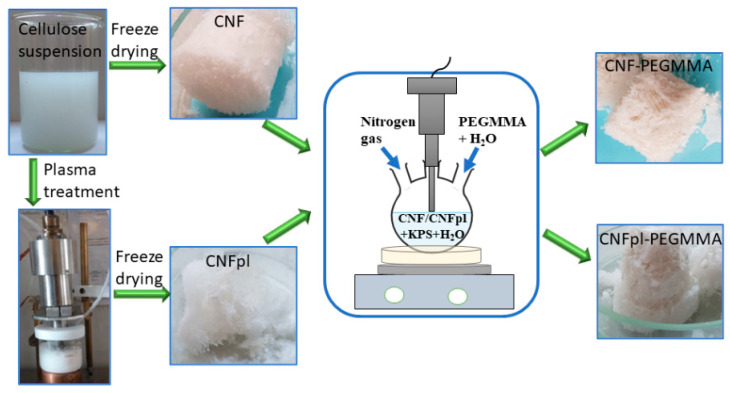
Schematic representation of the sample preparation.

The conditions of the synthesis have been described previously [15]. Briefly, 200 g of CNF suspension (0.065 mol/L) and 0.03 g KPS were added to a 250 mL three-necked round-bottom flask and stirred for 10 min under a nitrogen atmosphere. Separately, 1.8 g monomer was mixed with 3 mL water in a test tube and purged with nitrogen gas for 20 min. Afterward, the monomer solution was added into the reaction flask and allowed to polymerize at 55 °C, for 5 h, under magnetic stirring. The resulting suspension was precipitated in 400 mL of acetone in order to remove the unreacted chemicals and oligomers with very small molecular weight, and the sediment was washed with distilled water under vacuum filtration. In a subsequent purification step for removing the homopolymer, the grafted unmodified and plasma-modified celluloses (CNF-PEGMMA, CNFpl-PEGMMA) were washed in a Soxhlet extractor with distilled water and dimethylformamide (DMF) 1:1, for 20 h, and finally, with distilled water at reflux, for 3 h. The obtained grafted cellulose suspension was filtered under vacuum, and the white precipitate was further freeze-dried in order to obtain porous samples. The preparation of the samples is displayed in Figure 1.

#### 2.2.3. Characterization

The contact angles were measured at room temperature with an optical contact angle meter (CAM 200, Biolin Scientific, Espoo, Finland). Films of each sponge sample were obtained through compression in a laboratory press without additional heat. The static CAs of the droplets of deionized water with a volume of 6 μL placed onto the surface of the films were measured five times at different locations.

Dynamic Mechanical Analysis (DMA) of the samples was carried out on a DMA Q800 (TA Instruments, New Castle, DE, USA). Measurements were made at 30 °C, in compression mode, using 8 × 14 mm sponge samples. All the sponges were compressed with a ramp force of 0.5 N/min, from 0.001 to 18 N.

Fourier transform infrared spectroscopy (FTIR)-ATR measurements were performed in the 400–4000 cm^−1^ wavenumber range by accumulating 30 repetitive scans in order to guarantee a good signal-to-noise ratio and high reproducibility with a resolution of 4 cm^−1^. Samples were characterized using a JASCO 6300 FT-IR spectrophotometer (JASCO Int. Co., Ltd., Tokyo, Japan) equipped with a Golden Gate ATR (diamond crystal) from Specac Ltd., London, UK.

The surface morphology was analyzed using NOVA NanoSEM 630 field emission scanning electron microscope (FEI Company, Hillsboro, OR, USA), operated in high vacuum at 10 kV accelerating voltage. Before the investigation, a thick gold layer was deposited on the surfaces of the samples by using a conventional sputtering instrument (AUTO 500 instrument, BOC Edwards, West Sussex, UK) for better image quality.

The architectural features of the materials were imaged using micro-CT analyses performed with a SkyScan 1272 high-resolution X-ray micro-tomograph (Bruker Micro-CT, Kontich, Belgium) on triplicate samples. 2D images were registered at a camera binning of 1 × 1 with a voltage of 55 kV and 160 µA beam current. Five frames, registered with a rotation step of 0.1° at an exposure of 850 ms were averaged. The pixel size was set at 2 µm. The projections were reconstructed using CT Recon software (Version 1.7.1.6) and subsequently visualized as 3D objects using CTVox software (Version 3.3.0r1403). The porosity of the materials was assessed using CT Analyzer (Version 1.17.7.2).

Changes in the crystalline structure and crystallinity of CNF after plasma treatment and grafting were analyzed by X-ray diffraction (XRD). An XRD diffractometer (Rigaku Smart Lab SE, Tokyo, Japan) operating with Cu-Kα radiation (X-ray wavelength, λ = 1.5418 Å) in parallel beam configuration over the 2θ range of 10–40° was used for this purpose. The degree of crystallinity (C) was calculated as a ratio between the areas under the crystalline peaks and the total area of crystalline and amorphous peaks [21,22]. The XRD patterns were deconvoluted with Gaussian–Lorentzian functions to fit the crystalline peaks using Fityk software [23]. The interplanar spacing (d-spacing) was calculated using Bragg’s law, and the crystallite size was calculated with the Scherrer equation [21,24].

The MTT (3-(4,5-dimethylthiazol-2-yl)-2,5-diphenyltetrazolium bromide)-test was employed in order to estimate the cytotoxicity of plasma-treated cellulose and acrylates grafted sponges. The test was performed using two different types of cells, namely L929 murine fibroblasts (ECACC, collection of Public Health England) and RAW 264.7 macrophages. Cells were grown in Dulbecco’s Modified Eagle Medium (DMEM, Lonza, Belgium) supplemented with 10% fetal bovine serum (FBS, Biochrom AG, Germany) and antibiotics, 100 U/mL penicillin–100 μg/mL streptomycin (Lonza, Belgium). The cells were seeded onto 96-well plates (2.5 × 10^4^ cells/well) and cultured overnight at 37 °C in a humidified atmosphere with 5% CO_2_. The cells were exposed to a series of sample extracts of different dilutions (100%, 50%, 25%, 12.5%, 6.25%, 3.13%, and 1.56%) for 24 h. The experiments were repeated three times. Cell viability was calculated considering that the control cells have 100% viability. Negative control (min. value) is growing media. All of the data are expressed as the means ± SD.

The evaluation of the pro-inflammatory effect using RAW 264.7 cells that were trypsinized, detached, and counted. After centrifugation at 200× *g* for 10 min, the cell pellet was resuspended, and 10^4^ cells/well were pipetted from the cell suspension onto 96-well plates. Cells were incubated in 100 μL complete DMEM medium/well. After 24 h, the medium was removed, fresh medium was added for the control, lipopolysaccharide (LPS) 10 µg/mL for the positive control, and materials extracted to investigate, making serial dilutions to obtain different final concentrations. In order to evidence the proinflammatory effect, TNF-α concentrations were determined in cell supernatants collected after 3 h of stimulation using an ELISA kit (DuoSet, R&D Systems Inc., Minneapolis, MN, USA) according to the manufacturer’s instructions. The experiments were performed in triplicate and repeated three times.

The significance of the differences between the two samples was determined using an unpaired Student’s t-test using Microsoft Excel 2016. Differences with *p*-values less than 0.05 were considered to be statistically significant.

## 3. Results and Discussion

### 3.1. Morphological Investigation

**Figure 2:**
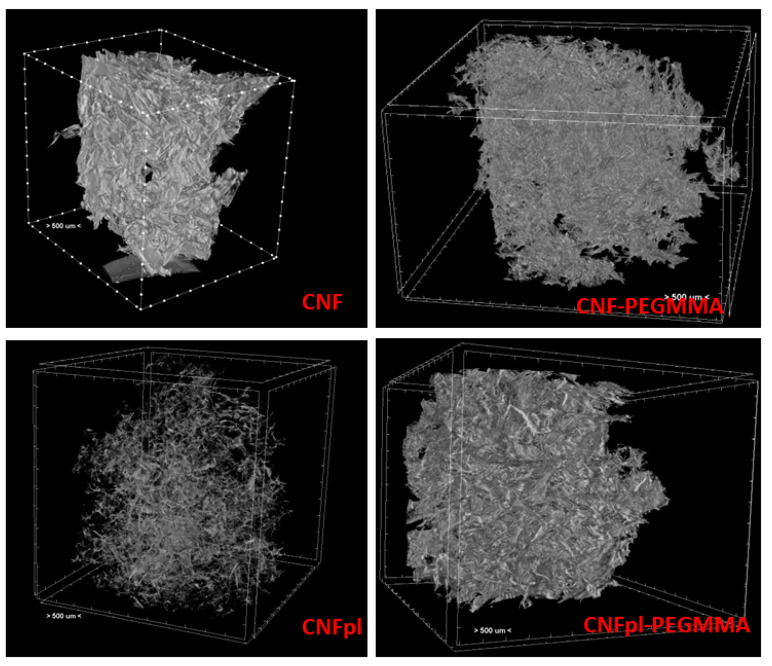
μ-CT images of ungrafted and PEGMMA-grafted CNF and CNFpl sponges.

**Figure 3:**
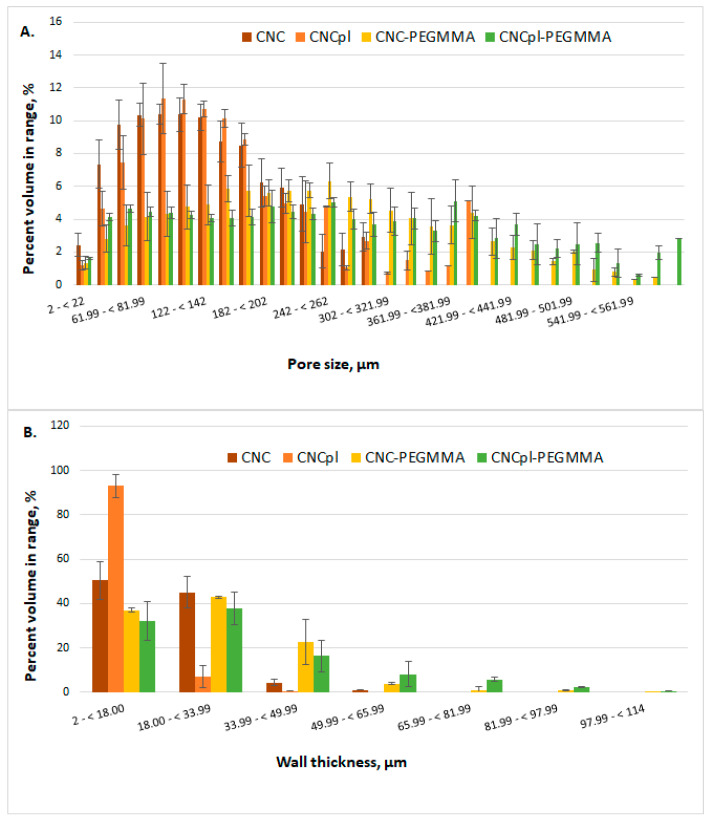
Pore size distribution (**A**) and wall thickness (**B**) in the analyzed scaffolds.

Neat and plasma-treated celluloses and their composites possess a sponge-like microstructure. Micro-computed tomography was used to obtain quantitative information on the three-dimensional structure of the cellulose sponges (Figure 2). Although all the sponges presented a porous structure after freeze-drying, the appearance of samples and their microstructure were different after plasma treatment and chemical modification: CNF microstructure consisted of large pores and agglomerated fibers, while that of CNPpl showed more individual nanofibers forming small bundles. Therefore, plasma treatment led to advanced defibrillation, also signaled in previous works [16,25], and prevented cellulose nanofibers from agglomerating. CNFpl sponge exhibited a small pore size similar to CNF but lower wall thickness than CNF due to defibrillation (Figure 3).

Important microstructural changes were observed after PEGMMA grafting, especially in the case of CNFpl, a more compact structure being observed for both CNF-PEGMMA and CNFpl-PEGMMA scaffolds (Figure 2). This supports successful grafting on both CNF and CNFpl.

Micro-CT analysis brings new information regarding the porous structure. As depicted in Figure 3A,B, native CNF has a porous architecture with small pores (smaller than 300 μm) and thin walls, most of them (95 vol%) up to 34 μm. The morphometric characteristics of CNFpl are similar in terms of pores sizes (90 vol% smaller than 300 μm) and wall thickness, which does not exceed 34 μm. However, a thorough analysis of wall thickness shows that 93 vol% was less than 18 μm in the case of CNFpl and only 50 vol% for CNF. The grafting of PEGMMA brings about significant changes in terms of microstructural features of both CNF and CNFpl-based scaffolds. The walls of the grafted scaffolds were thicker, especially in the case of CNFpl-PEGMMA, which had over 16 vol% of the walls in the thickness interval 50–114 μm compared with only 5 vol% in the case of CNF-PEGMMA. Moreover, the porosity of the scaffolds was substantially influenced by the grafting process. Porosity represents the percentage of void space and is calculated by dividing the volume of voids by the total volume [26]. In tissue engineering, porosity and pores interconnectivity are extremely important as they facilitate cell attachment and proliferation. It is worth remarking that total porosity increased from 89.3 ± 1.7 for CNF to 94.9 ± 1.1 for CNFpl, 91.3 ± 0.9 for CNC-PEGMMA and 91.7 ± 1.1 for CNCpl-PEGMMA. Both CNF-PEGMMA and CNFpl-PEGMMA scaffolds had a considerable number of larger pores when compared with the non-grafted counterparts. As opposed to CNF, which showed no pores in the interval 300–600 μm, CNF-PEGMMA had 33 vol% porosity in this interval. Similarly, CNFpl–based scaffolds exhibited less than 10 vol% of the pores in the interval 300–600 μm, while its grafted counterpart showed over 43 vol% in this interval.

**Figure 4:**
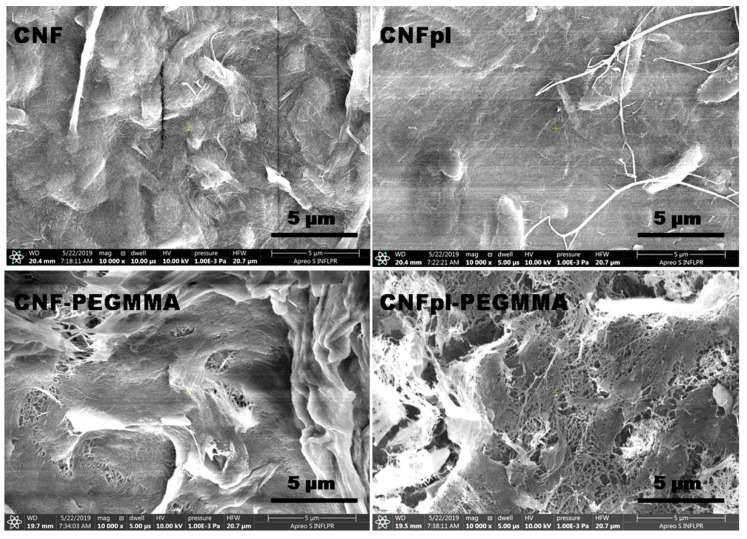
SEM images of CNF, CNF-PEGMMA, CNFpl and CNFpl-PEGMMA.

SEM images of CNF and modified CNFs (Figure 4) give more information on the morphological features at submicron level. The magnified SEM images reveal both individual and agglomerated cellulose nanofibers, similar to previous observations [15]. However, longer sparse fibers, detached from the sheets, can be frequently seen in the case of CNFpl, in good agreement with CT results. As previously reported, plasma treatment causes fiber breaking and defibrillation, resulting in thinner and detached fibers [25].

The presence of pores and cavities in the composite sponges increased significantly compared with CNF and CNFpl, showing the influence of the coating of cellulose nanofibers with more hydrophobic polymer blocks. This effect was more pronounced after plasma treatment, indicating an efficient grafting of the CNFpl surface as a result of the enhanced defibrillation. Most probably, the hydrophobic polymer coating reduced the proportion of bound water and increased the proportion of free water, leading to increased pore sizes. In addition, sheets of cellulose tightly bound by the polymer with no visible pores at this magnification were frequently observed on the surface of both composite sponges.

### 3.2. Structural Investigation

#### 3.2.1. FTIR Spectroscopy

**Figure 5:**
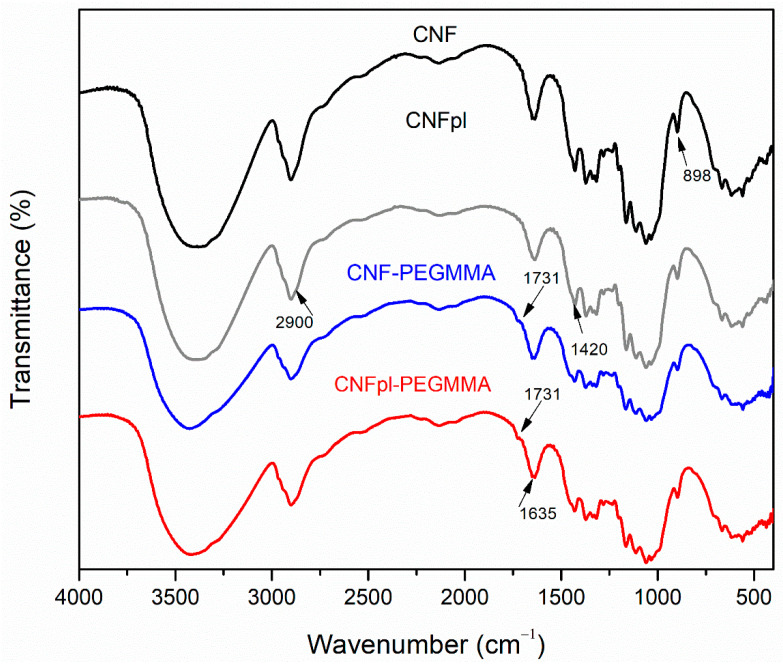
FTIR spectra of CNF and CNFpl sponges, before and after grafting reactions.

FTIR analysis revealed that changes occurred on the surface of CNF after plasma treatment and grafting reaction (Figure 5). Similar to non-treated CNF, FTIR spectra of all treated celluloses presented a high-intensity band between 3000 and 3600 cm^−1^, corresponding to the stretching vibration of abundant OH in the polysaccharide [25].

The peak at 2900 cm^−1^ is assigned to CH stretching vibrations, commonly used to estimate the crystallinity in cellulose-based materials. Grafting reactions determined a reduction in the intensity of this band, regardless of the type of cellulose. Although the cellulose peaks are predominant in the FTIR spectra and interfered with the characteristic peaks of acrylate functional groups, new shoulders were observed at 1731 cm^−1^ in CNF-PEGMMA and CNFpl-PEGMMA. This proves the presence of C=O stretching vibration of the ester bond in the composite sponges [27] and, therefore, the deposition of polymer onto the surface of cellulose nanofibers. Another characteristic band is around 1420 cm^−1^, attributed to CH_2_ deformation vibrations, which is useful for identifying the type of crystalline cellulose. Changes can be observed in the intensity of the peak, suggesting a variation in the degree of crystallinity of CNF after the treatments, similar to our previous report [25]. The peaks at 1370 cm^−1^ and 1317 cm^−1^ are attributed to the deformation vibrations of the C–H groups [28] and CH_2_ wagging [29], respectively. The following peaks, between 1170–920 cm^−1^, are related to the formation of different glycosidic linkages in polysaccharide [30], while the peak around 898 cm^−1^ [31] is used to differentiate the α and β configuration of anomeric carbon.

#### 3.2.2. Contact Angle Analysis

**Figure 6:**
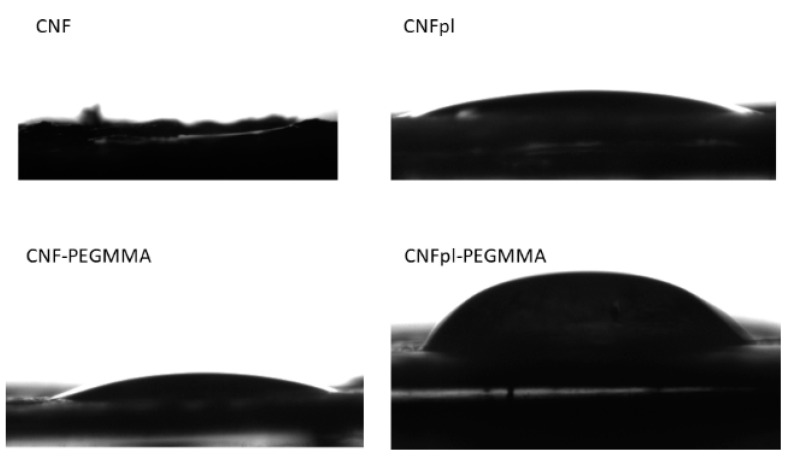
Water droplets on the surface of CNF, CNFpl, CNF-PEGMMA, and CNFpl-PEGMMA during the contact angle measurements.

Furthermore, we carried out contact angle measurements on all samples to gain information about the wetting characteristics of the sponges and to predict their behavior in aqueous liquids. A zero contact angle was found for the neat cellulose-compressed film with the addition of a water droplet, which was absorbed instantaneously in the pores (Figure 6). A measurable CA value of 11° was obtained for CNFpl, probably determined by the oxidative effect of air plasma treatment, which decreased the secondary alcoholic groups [32]. After the grafting reaction with PEGMMA, the water contact angle increased to 14° and 42° for CNF-PEGMMA and CNFpl-PEGMMA, respectively. PEGMMA can increase the hydrophobicity of the composite sample to a certain extent, possibly due to the aliphatic chains and ester bonds. These results are supported by FTIR analysis, which indicated the presence of carbonyl groups on the surface of the cellulose after grafting, which plays an important role in the decreased hydrophilic effect. Another explanation may be related to the more compact structure provided by the grafted polymer to cellulose sponges.

#### 3.2.3. Investigation of the Crystalline Structure by XRD

**Figure 7:**
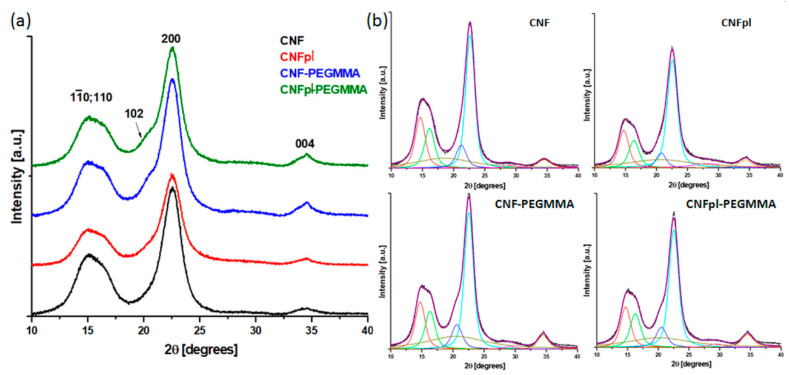
XRD diffractograms of CNF, CNFp, and their grafted counterparts (**a**); deconvolution of XRD patterns for unmodified and modified celluloses (**b**).

XRD patterns of cellulose before and after plasma and grafting treatments are shown in Figure 7. The XRD patterns show that the treatments applied to CNF did not change its crystalline structure, corresponding to the monoclinic unit cell of cellulose Iβ [33]. The diffraction peaks appeared at 2θ 14.7°, 16.3°, 21.1°, 22.6° and 34.5° in all the samples and correspond to the crystallographic planes (1ī0), (110), (102), (200), and (004), respectively [21,33].

The degree of crystallinity (C), d-spacing (d), and crystallite size (D) perpendicular to (110) and (200) crystal planes are given in Table 1. The values of C, d, and D, obtained for unmodified and modified CNFs, are similar to those obtained for microcrystalline cellulose subjected to various treatments [21,33]. The XRD parameters in Table 1 show that CNF treatments determined no significant change in the interplanar distance; however, a slight decrease in the crystallite size was noticed after plasma treatment or plasma activation and PEGMMA grafting. This is proof of the effectiveness of the treatments in the case of CNFpl and CNFpl-PEGMMA [33,34].

Significantly, plasma treatment and PEGMMA grafting led to a decrease in the degree of crystallinity of CNF (Table 1). The decrease in C value after plasma treatment could be explained by the morphological and structural changes induced by plasma activation, which promoted mechanical stresses and defibrillation along with CNF surface oxidation [25,33]. The changes in crystallinity after PEGMMA grafting were induced by the presence of the amorphous grafts and the reduction of hydrogen bonding due to the bonding with PEGMMA chains. This is consistent with SEM investigation results showing the breaking of fibers and defibrillation after plasma treatment and contact angle measurements indicating an increase in contact angle after plasma and PEGMMA treatment. Resuming the decrease in crystal size and crystallinity after the treatments and, especially after plasma treatment and plasma activation -PEGMMA grafting, is good evidence of the successful modification of CNF.

### 3.3. Thermal Stability

**Figure 8:**
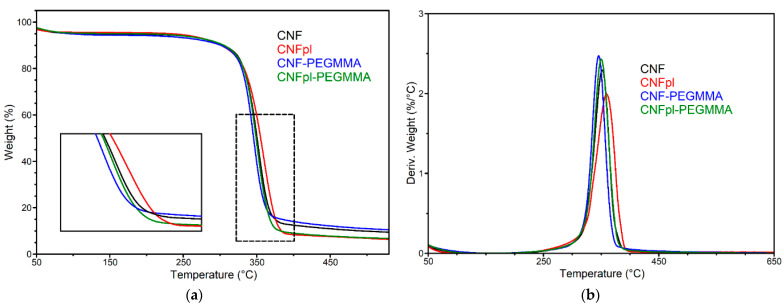
(**a**) TGA and (**b**) DTG curves for CNF and plasma treated cellulose and the corresponding grafted acrylate sponges.

The treatment of cellulose suspension in water using cold plasma and the grafting reaction of acrylates may influence the thermal stability of cellulose. The degradation of the original and modified cellulose sponges was a single-step process, as can be seen in TGA and derivative curves (Figure 8). The initial weight loss, up to 5%, is related to moisture loss. Both CNFpl and CNFpl-PEGMMA had much less moisture than their untreated counterparts, which is a result of plasma treatment that favored the release of bound water. The onset degradation temperature was around 331 °C, with small differences between cellulose sponges and the corresponding composites (Table 2). The chemical modification that allowed the formation of a hydrophobic coating on cellulose fiber did not significantly influence the thermal stability of cellulose. The temperature at the maximum degradation rate (T_max_) was around 350 °C for all the sponges, which can be linked to the α-cellulose degradation [35]. The grafting of PEGMMA slightly moved the degradation to lower temperatures compared with pure and plasma-treated celluloses. The decrease of T_max_ of CNFpl-PEGMMA as compared with its corresponding cellulose was higher than that observed for the CNF/CNF-PEGMMA, although the T_max_ of CNFpl-PEGMMA remained close to that of neat CNF. This may be attributed to the presence of a more substantial polyacrylate coating on the surface of the cellulose nanofibers as a consequence of the plasma treatment. The residue at 700 °C (R_700 °C_) was lower in the case of plasma-treated sponges, probably due to the effect of plasma causing the breaking of fibers and surface activation.

### 3.4. Mechanical Properties

**Figure 9:**
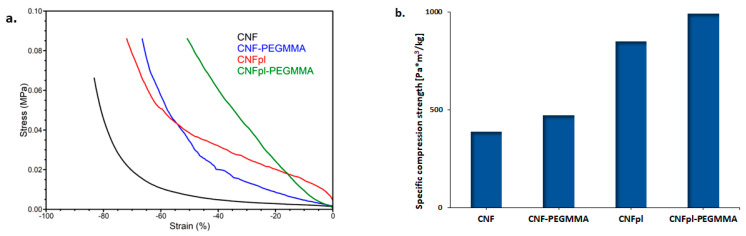
The compression stress-strain curves (**a**) and the specific compression strength of each sponge (**b**).

The mechanical properties of all the sponges are shown in Figure 9. The presence of PEGMMA on the cellulose backbone significantly changed the sponge characteristics leading to a stiffer material, as shown by the compression stress-strain curves from Figure 9a. In particular, the compression strength at 50% strain of CNF-PEGMA was 34 kPa, almost five times higher than that corresponding to unmodified CNF (7 kPa). Further, plasma treatment facilitated the preparation of a much stiffer composite, with a compression strength at a 50% strain value of 84 kPa. This value is much higher than that reported for gellan gum spongy-like hydrogels designed for tissue engineering and regenerative medicine (TERM) purpose [36]. Similarly, this increase in compression strength is more important than that obtained for nanofibrillated cellulose grafted with ethylene glycol methyl ether acrylate oligomer, where a three times increase in the compression strength values at 50% strain compared with ungrafted cellulose was reported [15]. Other work has shown that chemical pretreatment and ultrasonic time have influenced the mechanical properties of microfibrillated poplar catkin fiber aerogels and reported a compression strength value of 22–27 kPa for the prepared aerogels [37]. Much lower compression strength values at 50% strain, less than 10 kPa, were more recently reported by Osorio et al. for surface-modified cellulose nanocrystals with sulphate and phosphate groups, chemically cross-linked with hydrazone, with potential applicability as bone tissue scaffold [38].

The mechanical properties of spongy materials are affected significantly by their density, which can be easily calculated by dividing the mass of a piece of sponge with a well-defined shape by its apparent volume. In order to diminish the effect of density, specific compression strength was calculated for unmodified and modified CNF sponges. The specific resilience values, calculated by dividing the compression strength at 50% strain by the density, indicated that grafting PEGMMA onto CNF and CNFpl increased the specific compression strength by 22% and 16%, respectively (Figure 9b). The much higher increase in compression strength was noticed for plasma-treated cellulose (120%) and its composite (110%), as compared with the untreated counterparts. Interestingly, the intense defibrillation of cellulose promoted by plasma treatment, which led to an increase in density, did not affect the mechanical resistance of the sponge. On the contrary, mechanical strength increased sharply; moreover, the composite was dimensionally very stable, as more PEGMMA radicals reached more cellulose nanofibers during the polymerization reaction.

### 3.5. Biocompatibility Assay

**Figure 10:**
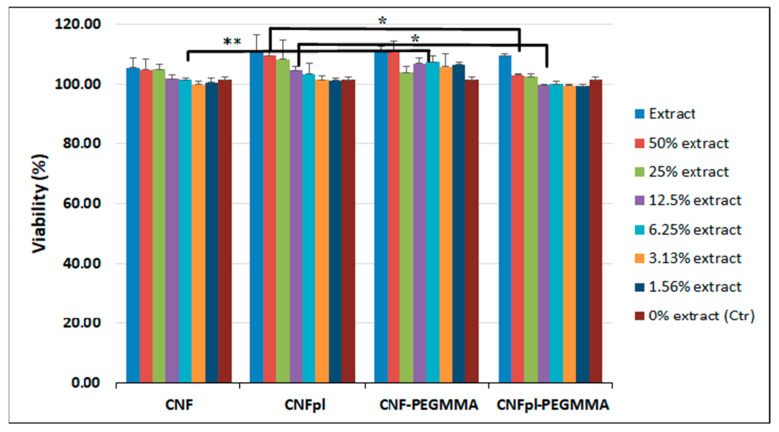
Cell viability measured by MTT test for CNF, CNFpl, CNF-PEGMMA, and CNFpl-PEGMMA sponges; data are the mean with error bars representing standard error of the mean over three independent experiments (** for *p* < 0.01; * for *p* < 0.05).

The cytotoxicity of CNF materials has been consistently investigated and nanocellulose have been proved to be safe and very useful in biomedical applications, for wound healing purpose, tissue regeneration, and repair, the composition of biosensors, for the development of drug delivery systems, among many other applications [39]. At the same time, PEG oligomers showed a certain cytotoxicity, more evident at a lower molecular weight and higher concentrations [40]. In this context, we examined the possible cytotoxic effect of CNF and CNF-derived sponges, which may be generated mostly by unreacted acrylate oligomers. Figure 10 displays the L929 cell viability by MTT assay at different sponge extract concentrations. The cell viability had values ranging from 99 to 111%, much higher in most cases than the control sample (101 %). Importantly, plasma treatment increased the cell viability of CNF, possibly due to new oxygen-containing groups generated on the surface of the cellulose fiber [41]. This effect was previously observed in the case of chitosan membranes [41] or poly((L-lactide)-co-(ϵ-caprolactone)) and poly((L-lactide)-co-glycolide) electrospun nanofibrous membranes [42]. Park et al. studied the in vitro cytotoxicity of plasma-induced nanostructured cellulose by using human foreskin fibroblast (Hs27) and immortalized human keratinocyte (HaCaT) cell lines, showing no cytotoxic effect on either cell type [43]. Remarkably, CNF-PEGMMA showed better cell viability, compared with the untreated CNF sample; this difference is statistically significant (*p* < 0.05). The surface modification of cellulose by plasma treatment, followed by grafting PEGMMA onto CNFpl, led to a slight decrease in cell viability; the difference is statistically significant (*p* < 0.05). Importantly, cell viability higher than 80% was obtained for all samples, which is considered a good viability [44]. This confirms that coating cellulose with PEGMMA blocks led to materials with good safety potential for biomedical applications.

**Figure 11:**
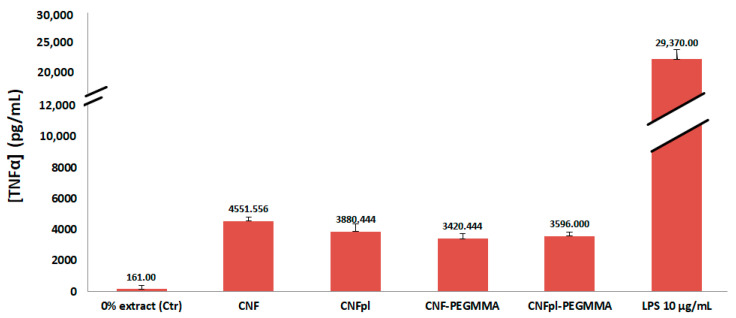
Release of TNF-α by RAW 264.7 murine macrophages after 24 h culture in CNF, CNFpl, CNF-PEGMMA, and CNFpl-PEGMMA sponges.

The inflammatory response of the macrophages was studied by the quantification of the pro-inflammatory cytokine TNF-α in the cell culture supernatants after 24 h. The experiments were performed in the presence of LPS to stimulate the pro-inflammatory environment. Results indicated that TNF-α secretion by cells in contact with CNFpl, CNF-PEGMMA, and CNFpl-PEGMMA samples was lower than that observed for the CNF sample (Figure 11). This demonstrates that the treatments applied to alter the surface properties of the cellulose nanofibers were properly designed to obtain materials able to suppress the inflammatory process. Particularly, the grafted cellulose sponges stand out as good candidates for use in biomedical applications and deserve further investigation.

## 4. Conclusions

Plasma treatment before CNF grafting with PEGMMA was found to be a feasible method to modify the original structural parameters, as evidenced by micro-CT and SEM analysis. Results showed that plasma treatment favored the defibrillation of cellulose nanofiber and enhanced the grafting of acrylate polymer chains. The porous structure of the CNF and CNFpl sponges became more compact with the addition of PEGMMA, showing larger pores (300–600 μm) and lower wall thickness when compared with the non-grafted counterparts. Grafting with PEGMMA increased the compression strength of both untreated and plasma-treated CNF. However the effect was much more important after plasma treatment. In addition, the grafting with PEGMMA reduced the hydrophilic character of CNF, especially after plasma pretreatment. Results showed that none of the cellulose-based sponges induces cytotoxicity. More than that, plasma treatment gave a higher viable cell numbers than pristine CNF. Considering the differences observed between CNFpl and CNFpl-PEGMMA and their corresponding untreated samples, we can consider that the mechanical strength, hydrophobicity, and cell viability were potentiated by the plasma treatment applied on cellulose nanofibers.

## Figures and Tables

**Table 1:** Parameters obtained from the deconvoluted XRD patterns: degree of crystallinity, d-spacing, and crystallite size perpendicular to (110) and (200) planes.

Sample	d_110_, nm	d_200_, nm	D_110_, nm	D_200_, nm	C, %
CNF	0.548	0.392	4.122	4.718	79.5
CNFpl	0.541	0.394	4.122	4.162	73.8
CNF-PEGMMA	0.543	0.394	4.198	4.717	75.0
CNFpl-PEGMMA	0.544	0.394	4.105	4.288	75.9

**Table 2:** Characteristic TGA temperatures (temperature at 5% weight loss—T_5%_, onset degradation temperature—T_onset_ and T_max_) and residue at 700 °C (R_700 °C_).

Sample	T_5%_ (°C)	T_onset_ (°C)	T_max_ (°C)	R_700 °C_
CNF	103.2	331.5	351.9	8.2
CNFpl	232.8	331.0	359.4	3.9
CNF-PEGMMA	95.7	328.6	345.7	9.2
CNFpl-PEGMMA	184.4	331.3	350.2	5.9

## Data Availability

The data presented in this study are available on request from the corresponding author.
